# Association Study between the *FTCDNL1* (*FONG) *and Susceptibility to Osteoporosis

**DOI:** 10.1371/journal.pone.0140549

**Published:** 2015-10-22

**Authors:** Hsing-Fang Lu, Kuo-Sheng Hung, Yu-Wen Hsu, Yu-Ting Tai, Lin-Shan Huang, Yu-Jia Wang, Henry Sung-Ching Wong, Yi-Hsiang Hsu, Wei-Chiao Chang

**Affiliations:** 1 Department of Clinical Pharmacy, Taipei Medical University, Taipei, Taiwan; 2 Department of Pharmacy, Taipei Medical University-Shuang Ho Hospital, Taipei, Taiwan; 3 Department of Neurosurgery, Taipei Medical University-Wan Fang Hospital, Taipei, Taiwan; 4 Master Program for Clinical Pharmacogenomics and Pharmacoproteomics, School of Pharmacy, Taipei Medical University, Taipei, Taiwan; 5 Department of Anesthesiology, Taipei Medical University, Taipei, Taiwan; 6 Broad Institute of MIT and Harvard, Cambridge, MA, United States of America; 7 Hebrew SeniorLife Institute for Aging Research, Harvard Medical School, Boston, MA, United States of America; University of Umea, SWEDEN

## Abstract

Osteoporosis is a systemic skeletal disease characterized by a decreased bone mineral density that results in an increased risk of fragility fractures. Previous studies indicated that genetic factors are involved in the pathogenesis of osteoporosis. Polymorphisms of the *FONG (FTCDNL1)* gene (rs7605378) were reported to be associated with the risk of osteoporosis in a Japanese population. To assess whether polymorphisms of the *FTCDNL1* gene contribute to the susceptibility and severity of osteoporosis in a Taiwanese population, 326 osteoporosis patients and 595 controls of a Taiwanese population were included in this study. Our results indicated that rs10203122 was significantly associated with osteoporosis susceptibility among female. Our findings provide evidence that rs10203122 in *FTCDNL1* is associated with a susceptibility to osteoporosis.

## Introduction

Osteoporosis is a systemic skeletal disease characterized by a decreased bone mineral density (BMD) and skeletal fragility, resulting in an increased risk of fragility fractures [1]. Over 9 million osteoporotic fractures occur worldwide, and of these approximately 2 million occur annually in the United States, incurring US$17 billion in direct annual costs [2]. The prevalence of osteoporosis in Taiwan is about 1.63% in males and 11.35% in female aged over 50 years [3]. Morbidity and mortality associated with the disease substantially increase as the population ages [4]. Bone metabolism is a dynamic process that is a balance between osteoclastic bone resorption and bone formation by osteoblasts. An estrogen deficiency, aging, nutritional deficiencies, and certain medications might contribute to bone loss and are correlated with the osteoporosis pathogenesis. Osteoporotic fractures are common and inflict substantial economic, social, and clinical burdens. Preventive therapies exist for fractures, and so individuals are screened by measuring the BMD.

Twin and family studies showed that BMD has a high heritability and estimated that 50%–85% of the variance in BMD is genetically determined [5–7]. BMD remains the single most clinically useful risk factor for osteoporotic fractures [8]. Previous genetic association studies reported that multiple genetic loci (*ESR1*, *LRP4*, *ITGA1*, *LRP5*, *SOST*, *SPP1*, *TNFRSF11A*, *TNFRSF11B*, and *TNFSF11*) were associated with BMD [9–11]. Furthermore, other groups conducted genome-wide association studies (GWASs) and identified single-nucleotide polymorphisms (SNPs) from many loci which were associated with BMD and osteoporotic fractures [12–16]. However, those studies all mainly focused on Caucasian populations. Recently, Kou *et al*. conducted a GWAS in Japan and identified a locus on chromosome 2q33.1 that showed a significant association with osteoporosis susceptibility [17]. This locus is in a previously unknown gene, which Kou *et al*. named *FONG* (formiminotransferase N-terminal sub-domain-containing gene). The gene is now formally named as formiminotransferase cyclodeaminase N-terminal like gene *(FTCDNL1)*.

The longest isoform of *FTCDNL1* gene is encoded a 147- amino-acid protein with a formiminotransferase domain in its N-terminal (FTCD_N domain) and is ubiquitously expressed in various tissues including bone. The FTCD-N domain has transferase activity that transfers a formimino group from N- formimino-L-glutamic acid to tetrahydrofolate to generate glutamic acid and 5-formiminotetrahydrofolate [18]. Glutamic acid is secreted by osteoclasts, and the signaling of glutamate is considered to have an important role in bone homeostasis. A previous study showed that knockout of the glutamate transporter 1 caused mice to develop osteoporosis [19]. Those results suggest that *FTCDNL1* plays a potential role in regulating bone metabolism. The purpose of this study was to replicate the findings from the GWAS conducted by Kou *et al*. in Japan and to determine which SNPs of *FTCDNL1* are associated with osteoporosis susceptibility via BMD T-scores and Z-scores in an Eastern Asian population at Taiwan.

## Material and Methods

### Patients and Methods

This study only included males and postmenopausal females aged ≥ 55 years. Patients with pathological fractures or high-impact fractures (such as those due to motor vehicle accidents) and continuous steroid user (of over 6 months) were excluded. We enrolled 326 cases that fulfilled the diagnostic criteria for osteoporosis and selected 595 controls without osteoporosis from Wan-Fang Hospital. Osteoporosis was diagnosed according to the criteria of the World Health Organization (WHO) of a T-score of ≤ -2.5 BMD was measured by dual energy radiograph absorptiometry with standard protocols at the lumbar spine (LS, L2-4 or L1-4) and/or femoral neck (FN). Vertebral fractures were assessed by digital measurements of morphologic changes on a lateral radiograph of the thoracolumbar spine. Subjects with a T-score (either at LS or FN) ≤ -2.5 were defined as the case group. We also collected clinical information such as age, gender, and a family history of osteoporosis. The study protocol conformed to the Declaration of Helsinki. The study was approved by the Joint Institutional Review Board of Taipei Medical University. All subjects provided written informed consent.

### DNA Extraction

DNA was extracted from whole blood samples collected from subjects. Blood cells were first treated with 0.5% sodium dodecylsulfate (SDS) lysis buffer and then with protease K (1 mg/ml) for 4 h at 60°C to digest the nuclear proteins. Total DNA was harvested using a Gentra (Qiagen, Valencia, CA) extraction kit and 70% alcohol precipitation, as described in our previous study [20].

### Genotyping

**Fig 1:**
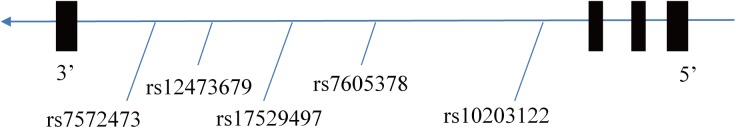
Graphical overview of the genotyped human *FTCDNL1* gene.

We selected five of the most significant SNPs (rs7572473, rs12473679, rs17529497, rs7605378, and rs10203122) of *FTCDNL1* based on a previous study with a minimum allele frequency of ≥ 1% in a Beijing Han Chinese population[17]. The *FTCDNL1* gene structure is shown in Fig 1. Genotyping was performed by the TaqMan Allelic Discrimination assay (Applied Biosystems, Foster City, CA). The polymerase chain reaction (PCR) was accomplished by an ABI StepOnePlus Thermal Cycler. In a subsequent PCR, the fluorescence from specific probes was detected and analyzed through the System SDS software version 2.2.2 (Applied Biosystems).

### Statistical Analysis

R 3.2.0 was used for the statistical analyses. The χ^2^-test was used to test whether minor allele frequencies in controls were deviated from Hardy-Weinberg equilibrium (HWE). A multiple logistic regression model was applied. Age and body mass index (BMI) were adjusted in the models as potential confounders. Associations between genotypes and risks of osteoporosis were tested by the likelihood ratio test. Odds ratios (ORs) and 95% confidence intervals (CIs) were estimated. In addition, we also performed an association analyses by multiple linear regressions with continue T-score and Z-score as the dependent variable. Since the definition of the Z-score is the subject BMD compared with the average BMD of the same-aged population, we did not consider age as a covariant in the Z-score. Pairwise linkage disequilibrium (LD) among genotyped SNPs was assessed and used to define haplotype blocks by the Haploview software v4.1. Haplotype analyses were also performed to examine associations between haplotypes and risks of osteoporosis by a general linear model (GLM) implemented in R. Since previous studies have shown the gender-specific genetic effect to osteoporosis risk [21], we analyzed men and women separately. To correct for multiple testing, false discovery rate (FDR) was applied and q-values were estimated to control for proper type I error. FDR q-values < 0.05 were considered statistical significance.

## Results

### Demographic and Clinical Characteristics of Subjects

In total, 326 osteoporosis cases and 595 controls were included in this study. All of the cases and controls are Taiwanese. The mean age ± standard deviation was 73.8±9.1 years for cases and 67.0±8.9 years for the control group. In all, 84.0% of osteoporosis patients and 72.4% of controls were female (Table 1).

**Table 1:** Basal characteristics of subjects.

	Cases (n = 326)		Controls (n = 595)	
	Male	Female	Male	Female
Sex	52 (16%)	274 (84%)	164 (28%)	431 (72 %)
Age (years)	75.6±9.96	73.5±9.0	69.9±9.56	65.9±8.4
BMI (kg/m^2^)	22.3±3.26	24.1±3.5	25.5±3.09	25.8±3.8

BMI, body mass index

### Associations between the *FTCDNL1* Genetic Polymorphisms and the Risk of Osteoporosis

All 5 genotyped SNPs (rs7572473, rs12473679, rs17529497, rs7605378, and rs10203122) were in Hardy-Weinberg equilibrium in controls (S1 Table). Among them, SNP rs10203122 was found to be significantly associated with osteoporosis in two genetic models (the genotype model: *q value = 0*.*04*; the recessive model: *q value = 0*.*01*) in the female population after adjusting for age and BMI (Table 2). SNP rs7605378 also showed significance under the “genotype” genetic model (*q value = 0*.*04*) after adjusting for age and BMI. However, no significant association of genotypes or allelic frequencies with osteoporosis susceptibility was found in the male population (S2 Table).

**Table 2:** Association analysis between *FTCDNL1* single-nucleotide polymorphisms and osteoporosis susceptibility in females.

		Number			Genotype		Dominant		Recessive		Allelic	
rs number	Genotype	Cases (%)	Controls (%)	OR (95%CI)	p value	q value	p value	q value	p value	q value	p value	q value
rs7572473	C/C	14(5.3%)	26(6.6%)	0.81(0.37–1.77)	0.415	0.519	0.409	0.607	0.444	0.554	0.707	0.790
	A/C	109(41.6%)	144(36.5%)	1.23(0.85–1.79)								
	A/A	139(53.1%)	224(56.9%)	1								
rs12473679	T/T	45(18.1%)	80(20.6%)	1.08(0.64–1.84)	0.955	0.955	0.884	0.884	0.764	0.764	0.790	0.790
	C/T	125(50.2%)	195(50.3%)	1.01(0.66–1.54)								
	C/C	79(31.7%)	113(29.1%)	1								
rs17529497	G/G	13(5.6%)	24(7%)	0.56(0.24–1.28)	0.374	0.519	0.486	0.607	0.171	0.285	0.269	0.673
	A/G	88(38.1%)	121(35.3%)	0.94(0.63–1.41)								
	A/A	130(56.3%)	198(57.7%)	1								
rs7605378	A/A	58(22.1%)	105(27%)	0.9(0.54–1.49)	**0.016**	**0.040**	0.185	0.464	0.052	0.129	0.719	0.790
	A/C	145(55.1%)	177(45.5%)	1.59(1.03–2.46)								
	C/C	60(22.8%)	107(27.5%)	1								
rs10203122	C/C	11(4.5%)	48(12.4%)	0.33(0.16–0.71)	**0.009**	**0.040**	0.139	0.464	**0.003**	**0.013**	**0.015**	0.077
	C/T	101(41.2%)	151(38.9%)	0.9(0.61–1.31)								
	T/T	133(54.3%)	189(48.7%)	1								

The p value was adjusted for age and the body-mass index. OR, odds ratio. CI, confidence interval. P-values and q-values < 0.05 are shown in bold. Q-values < 0.05 are considered statistical significance after correction for multiple testing.

### Associations between *FTCDNL1* Genetic Polymorphisms and BMD T-Scores and Z-Scores at LS and FN Skeletal Sites

We also analyzed the association between genetic polymorphisms of *FTCDNL1* and continue BMD measurements, including T-scores and Z-scores at LS and FN. In the female population, SNP rs10203122 was strongly associated with T-scores in both the recessive model (*p = 0*.*027*) and allelic model (*p = 0*.*026*). However, it was not achieved statistical significance after multiple testing corrections. The mean value of the T-score ± standard error of the SNP rs10203122 CC genotype was -1.67±0.12, which was higher than patients with the CT (-2.07±0.07) or TT (-2.12±0.06) genotypes (Table 3). Furthermore, SNP rs10203122 was also found to be strongly associated with Z-scores in the female population as the *p*-value was 0.049 when adjusted for the BMI (Table 4). However, it was not achieved statistical significance after multiple testing corrections. Although SNP rs7605378 was strongly associated with T-scores under the allelic model (*p = 0*.*042*) in the male population, it was not achieved statistical significance after multiple testing correction. Except for SNP rs7605378, none of the other SNPs associated with T-scores or Z-scores in the male population with p-values < 0.05 (S2 and S3 Tables).

**Table 3:** Association analysis between *FTCDNL1* single-nucleotide polymorphisms and T-scores in females.

			Value		Genotype		Dominant		Recessive		Allelic	
rs number	Genotype	Number	Mean	SE	p value	q value	p value	q value	p value	q value	p value	q value
rs7572473	C/C	40	-2.065	0.178	0.822	0.822	0.549	0.549	0.995	0.995	0.627	0.941
	A/C	253	-2.118	0.073								
	A/A	363	-2.053	0.058								
rs12473679	T/T	125	-1.999	0.097	0.310	0.388	0.376	0.470	0.370	0.462	0.941	0.941
	C/T	320	-1.998	0.065								
	C/C	192	-2.166	0.082								
rs17529497	G/G	37	-1.996	0.151	0.239	0.388	0.292	0.470	0.114	0.274	0.139	0.347
	A/G	209	-2.129	0.085								
	A/A	328	-2.078	0.060								
rs7605378	A/A	163	-2.020	0.082	0.093	0.231	0.268	0.470	0.165	0.274	0.868	0.941
	A/C	322	-2.158	0.069								
	C/C	167	-2.003	0.076								
rs10203122	C/C	59	-1.673	0.121	0.054	0.231	0.104	0.470	**0.027**	0.133	**0.026**	0.131
	C/T	252	-2.065	0.074								
	T/T	322	-2.117	0.063								

The p value was adjusted for age and the body-mass index. P-values and q-values < 0.05 are shown in bold. Q-values < 0.05 are considered statistical significance after correction for multiple testing.

**Table 4:** Association analysis between *FTCDNL1* single-nucleotide polymorphisms and Z-scores in females.

			Value		Genotype		Dominant		Recessive		Allelic	
rs number	Genotype	Number	Mean	SE	p value	q value	p value	q value	p value	q value	p value	q value
rs7572473	C/C	41	-0.142	0.122	0.995	0.995	0.998	0.998	0.924	0.924	0.968	0.972
	A/C	250	-0.123	0.059								
	A/A	359	-0.132	0.048								
rs12473679	T/T	126	-0.176	0.078	0.345	0.575	0.449	0.758	0.350	0.596	0.972	0.972
	C/T	317	-0.070	0.052								
	C/C	189	-0.165	0.068								
rs17529497	G/G	35	-0.011	0.135	0.655	0.818	0.826	0.998	0.358	0.596	0.589	0.972
	A/G	207	-0.147	0.067								
	A/A	326	-0.136	0.051								
rs7605378	A/A	164	-0.103	0.064	0.245	0.575	0.208	0.758	0.543	0.679	0.690	0.972
	A/C	316	-0.198	0.055								
	C/C	166	-0.065	0.070								
rs10203122	C/C	59	0.109	0.112	0.144	0.575	0.455	0.758	**0.049**	0.246	0.149	0.744
	C/T	252	-0.147	0.059								
	T/T	317	-0.156	0.052								

The p value was adjusted for age and the body-mass index. P-values and q-values < 0.05 are shown in bold. Q-values < 0.05 are considered statistical significance after correction for multiple testing.

Haplotype associations between *FTCDNL1* haplotypes and osteoporosis risk

**Fig 2:**
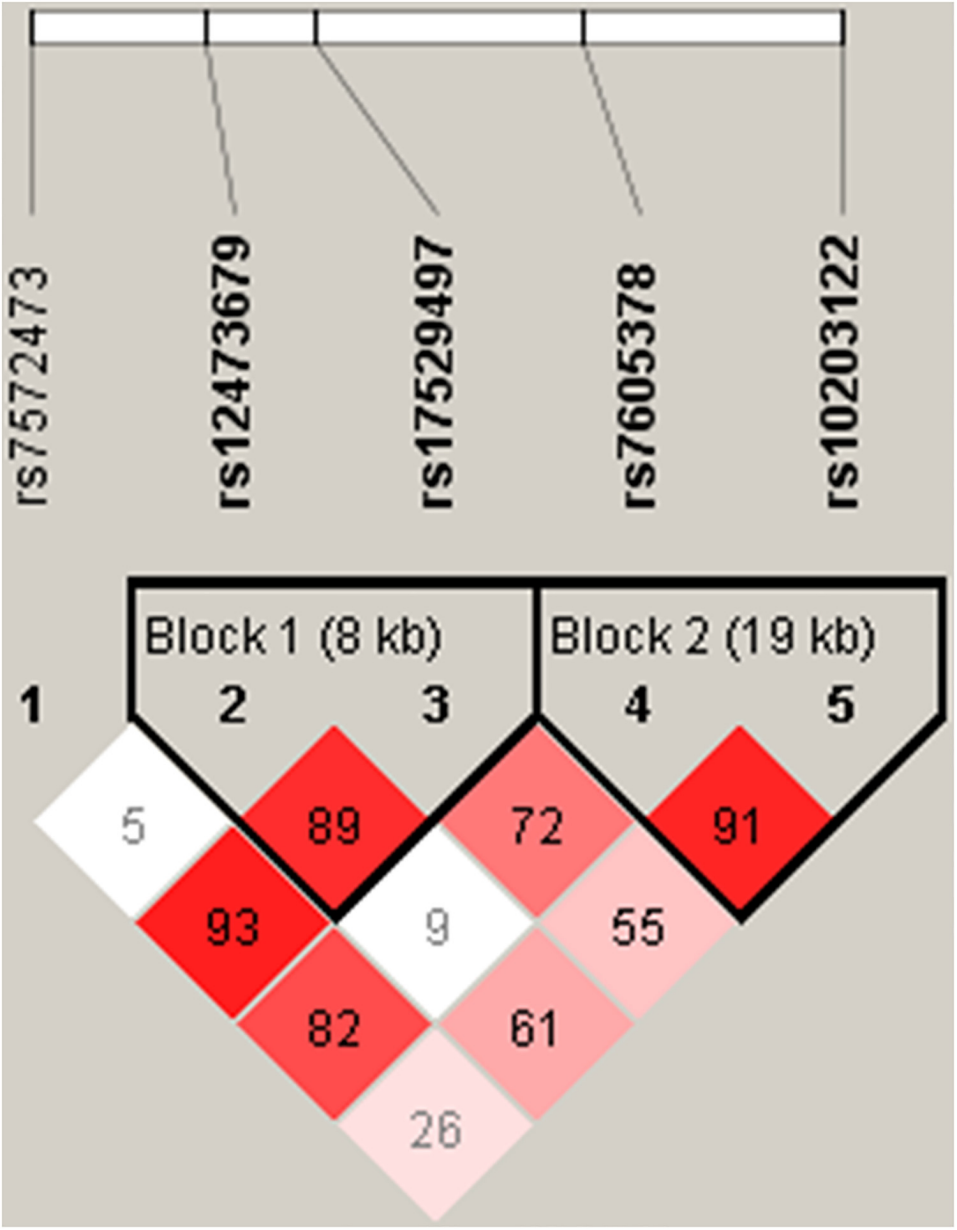
*FTCDNL1* gene linkage disequilibrium and haplotype block structure in osteoporosis. The number on the cell is the D’ (D’ x 100).

The result of pair-wise linkage disequilibrium of genotyped SNPs is shown in Fig 2. Two haplotype blocks are observed. We performed haplotype association analyses in each haplotype block, separately. In the haplotype block 2 (haplotypes formed by rs7605378 and rs10203122), compared to the reference C-T haplotype (rs7605378-rs10203122), the A-C haplotype is strongly associated with a lower risk of having osteoporosis (OR = 0.75, 95%CI = 0.60 ~ 0.95, p value = 0.016). However, after multiple testing corrections the result was not achieved statistical significance. Other haplotype association analysis results are shown in Table 5. No association was found in the haplotype block 1.

**Table 5:** Haplotype frequencies of the *FTCDNL1* gene in controls and patients with osteoporosis.

	Frequency				
	Case	Control	OR (95% CI)	*p* value	*q* value
**Block 1 rs12473679/rs17529497**					
CA	0.342	0.305	1.16 (0.92 ~1.48)	0.209	0.345
CG	0.239	0.243	1.09 (0.85 ~ 1.41)	0.489	0.489
TG	0.006	0.014	0.48 (0.10 ~2.28)	0.354	0.425
TA	0.409	0.444	Reference		
**Block 2 rs7605378/rs10203122**					
AT	0.243	0.198	1.17 (0.91 ~ 1.51)	0.230	0.345
CC	0.007	0.015	0.40 (0.11 ~ 1.43)	0.160	0.345
AC	0.246	0.303	0.75 (0.60 ~ 0.95)	**0.016**	0.094
CT	0.505	0.484	Reference		

P-value or Q-value < 0.05 are shown in bold. OR, odds ratio. CI, confidence interval.

## Discussion

We have identified that SNPs rs10203122 and rs7605378, two non-coding SNPs located in the intron3 of *FTCDNL1* gene, were associated with a lower risk of osteoporosis. We used F-SNP program (compbio.cs.queensu.ca/F-SNP/) to predict functional effects of the associated SNP with respect to the functional categories such as protein coding, splicing regulation, transcriptional regulation, and post-translation [22]. In silico analysis of the functional prediction of rs10203122 indicated that this SNP belonged to the transcriptional regulation category with functional score (FS) of 0.101. However, SNP rs10203122 was not mapped to any regulatory elements estimented by ENCODE and Roadmap Epigenomics projects by searching the HaploReg browser (www.broadinstitute.org/mammals/haploreg). Alternatively, SNP rs7605278 were located in histone binding alterations and might cause motif changes in HUES48 Cell Line (Human Embryonic Stem (HUES) Cell), suggesting that this SNP may involve in the regulation of *FTCDNL1* expression. To discover the association between gene expression profile and SNPS rs10203122 and rs7605378, we searched GTEx Portal (http://www.gtexportal.org/home/) which include a variety of tissues eQTL except bone-related tissue. There is no significant association profile of these two SNPs with gene expression in non-bone related tissue.

Consistent with a published GWAS in a Japanese sample, polymorphisms in *FTCDNL1* gene region were associated with a decreased risk of having osteoporosis in our study in a Taiwanese sample, suggesting this gene may be involved in the pathogenesis of osteoporosis. In the GWAS in Japanese, the SNP rs7605378 was found to be the most significant SNP in the *FTCDNL1* gene locus and the minor allele was associated with a decreased risk of having osteoporosis [17]. On the other hand, the association between rs7605378 and the risk of osteoporosis was also observed in our study, but the association did not achieved statistical significance. It may be due to limited sample size with smaller statistical power in our study. Our study identified that only the SNP rs10203122 achieves statistical significance. The minor allele of rs10203122 is associated with risk to osteoporosis in Japanese GWAS. In the contrary, the effect of this SNP was opposite in our study. Since the r^2^ value of LD block rs10203122-rs7605378 is low (our study: r^2^ = 0.35; result in Kou *et al*.: r^2^ = 0.43), the causal/functional variant may not have the same association between rs10203122 and rs7605378. The minor allele frequencies of the SNP rs7605378 as well as other four genotyped SNPs are all similar between Taiwanese samples and Japanese populations (S1 Table). SNP rs7605378 and rs10203122 are in LD (D’ = 0.91; r^2^ = 0.35). To identify which one is the potential causal SNP if assuming one of the SNPs is causal, we conducted a conditional analysis by putting both SNP rs7605378 and SNP rs10203122 into a multiple regression model. We found SNP rs10203122 becomes non-significance in all of the genetic model. However, minor allele of SNP rs7605378 revealed strongly association with lower risk of osteoporosis, higher T-score and Z-score, which consistent with GWAS in Japan (S5 and S6 Tables). These results suggest that both SNPs are probably in high LD with the causal SNP(s) that were not genotyped, and SNP rs7605378 is probably more closed relevant to the causal variant. The underlying genetic model of the causal variant is probably in the allelic model. In addition, to find out if there are synergistic effects between these two SNPs, we performed haplotype analyses. However, it did not seem to have synergistic effects between these two SNPs. Both SNPs together did not have a stronger effect comparing to the effect of each SNP. The most significantly associated haplotype is the A-C haplotypes of SNP rs7605378-rs10203122, suggesting A-C haplotype make harbors the causal variant(s). To compare our findings to the findings in Caucasian samples, we searched results from a published GWAS meta-analysis from the GEFOS consortium [16]. Similar to our results, SNP rs7605378 and rs10203122 did not statistically associated with BMD in Caucasian samples (S6 Table). Since genome-wide association analyses with osteoporosis risk were not performed by the GEFOS consortium, we were not able to compare the results. In addition, since the minor allele frequencies of SNP rs7605378 and rs10203122 are quite different between our Taiwanese sample and GEFOS’s Caucasian sample, suggesting *FTCDNL1* locus may be race/ethnic-specific. Furthermore, the environment and life style may be distinct among different geographic regions/race/ethnicities, which may cause the difficulty to compare across various ethnicities.

Several potential limitations include: first, the osteoporotic fracture data of patients was not collected. Thus, we were not able to examine the correlations between genetic polymorphisms and clinical outcomes, such as osteoporosis fractures. Second, the modest sample size (921 subjects) limited our statistical power. Although our samples are homogeneous and well defined in terms of phenotype assessment, increasing sample size will allow us to detect modest effect size that most of the common SNPs have. Third, given our original goal was to replicate findings from a Japanese study, since we identified a different SNP associated with osteoporosis risk, after conditional analysis our results reveals consistent with Japanese study. Therefore, an independent Taiwanese sample is needed to replicate our findings. Fourth, we did not comprehensively survey and genotype all potential sequence variants in study samples, which would allow us to perform direct association to find potential causal variants. Targeted sequencing in the *FTCDNL1* locus in subjects carried A-C haplotypes of SNP rs7605378-rs10203122 may be helpful to identify the potential causal variants. Fifth, we did not find SNP rs10203122 located in bone cell-specific regulatory elements due to the limited data available for skeleton relevant cells and tissues. Certainly, we did not rule out that the non-coding SNP, rs10203122, is functional. Further cellular or animal experiments in relevant cells and tissues may be needed to characterize its function.

## Conclusion

In summary, our studies confirmed the association of the *FTCDNL1* SNPs and the risk of having osteoporosis. Polymorphisms of the *FTCHNL1* gene are associated with a reduced risk of having osteoporosis, suggesting a potential therapeutic target for osteoporosis.

## Supporting Information

S1 Fig *FTCDNL1* gene linkage disequilibrium and haplotype block structure in osteoporosis with r^2^ value. (DOCX)

S1 Table The basic characteristics of the SNPs. (DOCX)

S2 Table Association analysis between *FTCDNL1* single-nucleotide polymorphisms (SNPs) and osteoporosis susceptibility in male. (DOCX)

S3 Table Association analysis between *FTCDNL1* single-nucleotide polymorphisms (SNPs) and T-score in male. (DOCX)

S4 Table Association analysis between *FTCDNL1* single-nucleotide polymorphisms (SNPs) and Z-score in male. (DOCX)

S5 Table Four genetic model of p value in osteoporosis after conditional analysis. (DOCX)

S6 Table Four genetic model of p value in T-score/Z-score after conditional analysis. (DOCX)

S7 Table Association results between *FTCFNL1* SNPs and BMD from GEFOS data. (DOCX)

S8 Table Results of p value for Hardy-Weinberg equilibrium. (DOCX)
