# Supplementary material for: Association Study between the FTCDNL1 (FONG) and Susceptibility to Osteoporosis
Source: PLoS One. 2015 Oct 22;10(10):e0140549. doi: 10.1371/journal.pone.0140549 (PMC4619591; doi:10.1371/journal.pone.0140549)
Supplement: S1 Fig — (DOCX) [file pone.0140549.s001.docx]

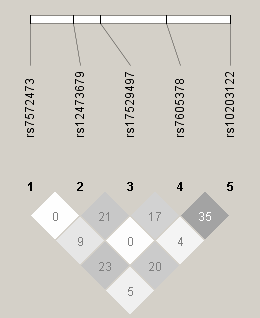
**S1 Fig. *FTCDNL1* gene linkage disequilibrium and haplotype block structure in osteoporosis with r^2^ value.** The number on the cell is the r^2^ (r^2^ x 100).
