# Supplementary material for: Association Study between the FTCDNL1 (FONG) and Susceptibility to Osteoporosis
Source: PLoS One. 2015 Oct 22;10(10):e0140549. doi: 10.1371/journal.pone.0140549 (PMC4619591; doi:10.1371/journal.pone.0140549)
Supplement: S1 Table — (DOCX) [file pone.0140549.s002.docx]

| **S1 Table. The basic characteristics of the SNPs.** | | | | | | | | | | | | |
| --- | --- | --- | --- | --- | --- | --- | --- | --- | --- | --- | --- | --- |
| **Gene** | **pos (hg38)** | **variant** | **Ref** | **Alt** | **AFR freq** | **AMR freq** | **ASN freq** | **EUR freq** | **JPT freq** | **TWB freq** | **Our study data** |  |
|  |  |  |  |  |  |  |  |  |  |  |  | **HWE** |
|  |  |  |  |  |  |  |  |  |  |  |  |  |
| *FONG* | chr2:199770194 | rs7572473 | C | A | 0.12 | 0.6 | 0.74 | 0.77 | 0.7 | 0.78 | 0.75 | 0.55 |
|  | chr2:199783496 | rs12473679 | T | C | 0.14 | 0.43 | 0.56 | 0.54 | 0.6 | 0.53 | 0.54 | 0.40 |
|  | chr2:199791834 | rs17529497 | A | G | 0.03 | 0.25 | 0.29 | 0.33 | 0.3 | 0.29 | 0.25 | 0.86 |
|  | chr2:199812203 | rs7605378 | A | C | 0.62 | 0.59 | 0.52 | 0.69 | 0.5 | 0.56 | 0.50 | 0.53 |
|  | chr2:199831723 | rs10203122 | T | C | 0.12 | 0.3 | 0.28 | 0.12 | 0.3 | 0.3 | 0.30 | 0.88 |
| Freq frequency shows as alt allele. TWB frequency based on Taiwan Biobank. HWE p value for Hardy-Weinberg equilibrium. | | | | | | | | | | | | |
