# Supplementary material for: Association Study between the FTCDNL1 (FONG) and Susceptibility to Osteoporosis
Source: PLoS One. 2015 Oct 22;10(10):e0140549. doi: 10.1371/journal.pone.0140549 (PMC4619591; doi:10.1371/journal.pone.0140549)
Supplement: S2 Table — (DOCX) [file pone.0140549.s003.docx]

| **S2 Table. Association analysis between *FTCDNL1* single-nucleotide polymorphisms (SNPs) and osteoporosis susceptibility in male.** | | | | | | | | | | | | | | | | |
| --- | --- | --- | --- | --- | --- | --- | --- | --- | --- | --- | --- | --- | --- | --- | --- | --- |
| **rs number** | **Genotype** | **Number** | | | | **OR** | **95%CI** | | **Genotype** | **Dominant** | **Recessive** | **Allelic** | **Genotype¶** | **Dominant¶** | **Recessive¶** | **Allelic¶** |
|  |  | **Case** | **(%)** | **Control** | **(%)** |  | **lower** | **upper** | ***P* Value** | ***P* Value** | ***P* Value** | ***P* Value** | ***P* Value** | ***P* Value** | ***P* Value** | ***P* Value** |
| rs7572473 | C/C | 1 | 2 | 8 | 5.3 | 0.54 | 0.06 | 4.8 | 0.5464 | 0.5446 | 0.3004 | 0.3901 | 0.7833 | 0.5692 | 0.5968 | 0.5056 |
|  | A/C | 18 | 35.3 | 55 | 36.2 | 0.84 | 0.4 | 1.78 |  |  |  |  |  |  |  |  |
|  | A/A | 32 | 62.7 | 89 | 58.6 | 1 |  |  |  |  |  |  |  |  |  |  |
| rs12473679 | T/T | 9 | 17.6 | 34 | 22.5 | 0.81 | 0.27 | 2.48 | 0.6780 | 0.9295 | 0.4183 | 0.6745 | 0.2642 | 0.4528 | 0.2543 | 0.8620 |
|  | C/T | 29 | 56.9 | 78 | 51.7 | 1.66 | 0.7 | 3.97 |  |  |  |  |  |  |  |  |
|  | C/C | 13 | 25.5 | 39 | 25.8 | 1 |  |  |  |  |  |  |  |  |  |  |
| rs17529497 | G/G | 2 | 4.4 | 6 | 4.3 | 0.54 | 0.08 | 3.82 | 0.8442 | 0.5644 | 0.9567 | 0.6086 | 0.6734 | 0.7522 | 0.4559 | 0.9938 |
|  | A/G | 20 | 44.4 | 55 | 39.9 | 1.21 | 0.56 | 2.6 |  |  |  |  |  |  |  |  |
|  | A/A | 23 | 51.1 | 77 | 55.8 | 1 |  |  |  |  |  |  |  |  |  |  |
| rs7605378 | A/A | 11 | 21.6 | 40 | 26.1 | 0.71 | 0.26 | 1.93 | 0.5039 | 0.2599 | 0.4829 | 0.2625 | 0.4315 | 0.2280 | 0.9889 | 0.4483 |
|  | A/C | 23 | 45.1 | 74 | 48.4 | 0.57 | 0.24 | 1.33 |  |  |  |  |  |  |  |  |
|  | C/C | 17 | 33.3 | 39 | 25.5 | 1 |  |  |  |  |  |  |  |  |  |  |
| rs10203122 | C/C | 3 | 6.5 | 12 | 7.7 | 1.1 | 0.26 | 4.67 | 0.4903 | 0.2328 | 0.7232 | 0.2647 | 0.6938 | 0.5061 | 0.7360 | 0.6895 |
|  | C/T | 19 | 41.3 | 77 | 49.4 | 0.73 | 0.33 | 1.6 |  |  |  |  |  |  |  |  |
|  | T/T | 24 | 52.2 | 67 | 42.9 | 1 |  |  |  |  |  |  |  |  |  |  |
| *¶P value adjusted for age and BMI. Significance shows in bold.* | | | | | | | | | | | | | | | | |
