# Supplementary material for: Association Study between the FTCDNL1 (FONG) and Susceptibility to Osteoporosis
Source: PLoS One. 2015 Oct 22;10(10):e0140549. doi: 10.1371/journal.pone.0140549 (PMC4619591; doi:10.1371/journal.pone.0140549)
Supplement: S3 Table — (DOCX) [file pone.0140549.s004.docx]

| **S3 Table. Association analysis between *FTCDNL1* single-nucleotide polymorphisms (SNPs) and T-score in male.** | | | | | | | | | | | | |
| --- | --- | --- | --- | --- | --- | --- | --- | --- | --- | --- | --- | --- |
| **rs number** | **Genotype** | **Number** | **Value** | | **Genotype** | **Dominant** | **Recessive** | **Allelic** | **Genotype¶** | **Dominant¶** | **Recessive¶** | **Allelic¶** |
|  |  |  | **Mean** | **SE** | ***P* Value** | ***P* Value** | ***P* Value** | ***P* Value** | ***P* Value** | ***P* Value** | ***P* Value** | ***P* Value** |
| rs7572473 | C/C | 9 | -1.4111 | 0.3706 | 0.8117 | 0.5698 | 0.6573 | 0.5225 | 0.8606 | 0.5899 | 0.9661 | 0.6373 |
|  | A/C | 73 | -1.5616 | 0.1268 |  |  |  |  |  |  |  |  |
|  | A/A | 121 | -1.6163 | 0.1011 |  |  |  |  |  |  |  |  |
| rs12473679 | T/T | 43 | -1.5133 | 0.1791 | 0.2774 | 0.1227 | 0.3736 | 0.1296 | 0.5600 | 0.4166 | 0.3551 | 0.2838 |
|  | C/T | 107 | -1.5589 | 0.1084 |  |  |  |  |  |  |  |  |
|  | C/C | 52 | -1.8115 | 0.1278 |  |  |  |  |  |  |  |  |
| rs17529497 | G/G | 8 | -1.9125 | 0.3681 | 0.3419 | 0.1925 | 0.3387 | 0.1457 | 0.5851 | 0.3026 | 0.7360 | 0.3145 |
|  | A/G | 75 | -1.6440 | 0.1260 |  |  |  |  |  |  |  |  |
|  | A/A | 100 | -1.4727 | 0.1103 |  |  |  |  |  |  |  |  |
| rs7605378 | A/A | 51 | -1.3608 | 0.1611 | 0.1240 | 0.1216 | 0.0726 | ***0.0421*** | 0.2204 | 0.1051 | 0.2395 | 0.0890 |
|  | A/C | 97 | -1.6010 | 0.1149 |  |  |  |  |  |  |  |  |
|  | C/C | 56 | -1.7584 | 0.1396 |  |  |  |  |  |  |  |  |
| rs10203122 | C/C | 15 | -1.1867 | 0.3152 | 0.1554 | 0.1092 | 0.1464 | 0.0566 | 0.5685 | 0.4422 | 0.3660 | 0.3155 |
|  | C/T | 96 | -1.4823 | 0.1156 |  |  |  |  |  |  |  |  |
|  | T/T | 91 | -1.6810 | 0.1093 |  |  |  |  |  |  |  |  |
| *¶P value adjusted for age and BMI. Significance shows in bold.* | | | | | | | | | | | | |
