# Supplementary material for: Association Study between the FTCDNL1 (FONG) and Susceptibility to Osteoporosis
Source: PLoS One. 2015 Oct 22;10(10):e0140549. doi: 10.1371/journal.pone.0140549 (PMC4619591; doi:10.1371/journal.pone.0140549)
Supplement: S4 Table — (DOCX) [file pone.0140549.s005.docx]

| **S4 Table. Association analysis between *FTCDNL1* single-nucleotide polymorphisms (SNPs) and Z-score in male.** | | | | | | | | | | | | |
| --- | --- | --- | --- | --- | --- | --- | --- | --- | --- | --- | --- | --- |
| **rs number** | **Genotype** | **Number** | **Value** | | **Genotype** | **Dominant** | **Recessive** | **Allelic** | **Genotype¶** | **Dominant¶** | **Recessive¶** | **Allelic¶** |
|  |  |  | **Mean** | **SE** | ***P* Value** | ***P* Value** | ***P* Value** | ***P* Value** | ***P* Value** | ***P* Value** | ***P* Value** | ***P* Value** |
| rs7572473 | C/C | 9 | -0.1778 | 0.2914 | 0.7701 | 0.4953 | 0.9520 | 0.5766 | 0.7007 | 0.4687 | 0.8194 | 0.5947 |
|  | A/C | 73 | -0.1068 | 0.1165 |  |  |  |  |  |  |  |  |
|  | A/A | 120 | -0.2125 | 0.0977 |  |  |  |  |  |  |  |  |
| rs12473679 | T/T | 42 | -0.1452 | 0.1839 | 0.6081 | 0.3457 | 0.5467 | 0.3356 | 0.8491 | 0.6263 | 0.6633 | 0.5674 |
|  | C/T | 107 | -0.1748 | 0.0994 |  |  |  |  |  |  |  |  |
|  | C/C | 52 | -0.3327 | 0.1116 |  |  |  |  |  |  |  |  |
| rs17529497 | G/G | 8 | -0.2750 | 0.2871 | 0.9048 | 0.7106 | 0.7410 | 0.6640 | 0.9942 | 0.9154 | 0.9665 | 0.9157 |
|  | A/G | 75 | -0.1747 | 0.1158 |  |  |  |  |  |  |  |  |
|  | A/A | 99 | -0.1535 | 0.1093 |  |  |  |  |  |  |  |  |
| rs7605378 | A/A | 51 | 0.0157 | 0.1426 | 0.2514 | 0.4675 | 0.0976 | 0.1488 | 0.5550 | 0.4727 | 0.3086 | 0.2932 |
|  | A/C | 97 | -0.2175 | 0.1131 |  |  |  |  |  |  |  |  |
|  | C/C | 55 | -0.2200 | 0.1236 |  |  |  |  |  |  |  |  |
| rs10203122 | C/C | 15 | -0.0667 | 0.2760 | 0.9063 | 0.7248 | 0.7246 | 0.6652 | 0.9998 | 0.9924 | 0.9853 | 0.9877 |
|  | C/T | 96 | -0.1188 | 0.1138 |  |  |  |  |  |  |  |  |
|  | T/T | 90 | -0.1744 | 0.1003 |  |  |  |  |  |  |  |  |
| *¶P value adjusted for age and BMI. Significance shows in bold.* | | | | | | | | | | | | |
