# Supplementary material for: Association Study between the FTCDNL1 (FONG) and Susceptibility to Osteoporosis
Source: PLoS One. 2015 Oct 22;10(10):e0140549. doi: 10.1371/journal.pone.0140549 (PMC4619591; doi:10.1371/journal.pone.0140549)
Supplement: S5 Table — (DOCX) [file pone.0140549.s006.docx]

| **S5 Table . Four genetic model of p value in osteoporosis risk after conditional analysis** | | | | | | | | | | | |
| --- | --- | --- | --- | --- | --- | --- | --- | --- | --- | --- | --- |
| SNP | Adjustment | Genotype | Case | % | Control | % | OR (95%CI) | Genotype | Dominant | Recessive | Allelic |
|  |  |  |  |  |  |  |  | p value | p value | p value | p value |
| rs7605378 | rs10203122 | C/C | 10 | 4.1 | 43 | 11.7 | 0.33(0.13-0.83) | 0.050 | 0.204 | **0.023** | **0.039** |
|  |  | C/T | 100 | 41.2 | 140 | 38 | 0.81(0.52-1.26) |  | 0.75(0.48-1.17) | 0.38(0.16-0.91) | 0.68(0.47-0.98) |
|  |  | T/T | 133 | 54.7 | 185 | 50.3 | 1 |  |  |  |  |
| rs10203122 | rs7605378 | A/A | 55 | 22.6 | 99 | 26.9 | 1.28(0.67-2.44) | 0.197 | 0.099 | 0.689 | 0.391 |
|  |  | A/C | 130 | 53.5 | 169 | 45.9 | 1.55(0.95-2.53) |  | 1.5(0.93-2.43) | 0.9(0.54-1.51) | 1.15(0.83-1.59) |
|  |  | C/C | 58 | 23.9 | 26 | 22.4 | 1 |  |  |  |  |
| The p value was adjusted for age and the body-mass index. P-values and q-values < 0.05 are shown in bold. | | | | | | | | | | | |
