# Supplementary material for: Association Study between the FTCDNL1 (FONG) and Susceptibility to Osteoporosis
Source: PLoS One. 2015 Oct 22;10(10):e0140549. doi: 10.1371/journal.pone.0140549 (PMC4619591; doi:10.1371/journal.pone.0140549)
Supplement: S6 Table — (DOCX) [file pone.0140549.s007.docx]

| **S6 Table. Four genetic model of p value in T-score/Z-score after conditional analysis** | | | | | | | | | | |
| --- | --- | --- | --- | --- | --- | --- | --- | --- | --- | --- |
|  |  |  |  |  | Value | | Genotype | Dominant | Recessive | Allelic |
|  | SNP | Condition on | Genotype | Number | Mean | SE | p value | p value | p value | p value |
| T-score | rs7605378 | rs10203122 | C/C | 53 | -1.675 | 0.131 | 0.065 | 0.068 | 0.082 | **0.022** |
|  |  |  | C/T | 240 | -2.087 | 0.076 |  |  |  |  |
|  |  |  | T/T | 318 | -2.130 | 0.063 |  |  |  |  |
|  | rs10203122 | rs7605378 | A/A | 154 | -2.025 | 0.084 | 0.240 | 0.096 | 0.971 | 0.260 |
|  |  |  | A/C | 299 | -2.128 | 0.073 |  |  |  |  |
|  |  |  | C/C | 158 | -2.017 | 0.079 |  |  |  |  |
| Z-score | rs7605378 | rs10203122 | C/C | 53 | 0.117 | 0.119 | 0.066 | 0.232 | **0.030** | **0.046** |
|  |  |  | C/T | 240 | -0.156 | 0.060 |  |  |  |  |
|  |  |  | T/T | 313 | -0.166 | 0.052 |  |  |  |  |
|  | rs10203122 | rs7605378 | A/A | 155 | -0.102 | 0.066 | 0.330 | 0.150 | 0.517 | 0.173 |
|  |  |  | A/C | 294 | -0.178 | 0.057 |  |  |  |  |
|  |  |  | C/C | 157 | -0.096 | 0.071 |  |  |  |  |

The p value was adjusted for age and the body-mass index. P-values and q-values < 0.05 are shown in bold.
