# Supplementary material for: Association Study between the FTCDNL1 (FONG) and Susceptibility to Osteoporosis
Source: PLoS One. 2015 Oct 22;10(10):e0140549. doi: 10.1371/journal.pone.0140549 (PMC4619591; doi:10.1371/journal.pone.0140549)
Supplement: S7 Table — (DOCX) [file pone.0140549.s008.docx]

| S7 Table. Association results between *FTCFNL1* SNPs and BMD from GEFOS data. | | | | | | | | | | | | |
| --- | --- | --- | --- | --- | --- | --- | --- | --- | --- | --- | --- | --- |
| Gene | rs number | GEFOS_LS female | | | | |  | GEFOS_FN female | | | | |
|  |  | Allele1 | Allele2 | P-value | Direction | Freq. |  | Allele1 | Allele2 | P-value | Direction | Freq. |
| FONG | rs7572473 | NA | | | | |  | NA | | | | |
|  | rs12473679 | NA | | | | |  | NA | | | | |
|  | rs17529497 | a | g | 0.6977 | + | 0.6833 |  | a | g | 0.7317 | - | 0.6833 |
|  | rs7605378 | a | c | 0.9184 | + | 0.3833 |  | a | c | 0.3652 | + | 0.3833 |
|  | rs10203122 | t | c | 0.8393 | + | 0.8500 |  | t | c | 0.9597 | - | 0.8500 |
| Frequency:Allele1.HapMapCEU | | | | | | | | | | | | |
