# Supplementary material for: Association Study between the FTCDNL1 (FONG) and Susceptibility to Osteoporosis
Source: PLoS One. 2015 Oct 22;10(10):e0140549. doi: 10.1371/journal.pone.0140549 (PMC4619591; doi:10.1371/journal.pone.0140549)
Supplement: S8 Table — (DOCX) [file pone.0140549.s009.docx]

| **S8 Table. Results of p value for Hardy-Weinberg equilibrium.** | | | | | | | | | | | | |
| --- | --- | --- | --- | --- | --- | --- | --- | --- | --- | --- | --- | --- |
|  | alleles | Male | | | | |  | Female | | | | |
|  |  | MAF(%) |  | HWE | | |  | MAF(%) |  | HWE | | |
|  |  |  |  | All | control | case |  |  |  | All | control | case |
| rs7572473 | A/C | 22.6 |  | 0.69 | 1.00 | 0.66 |  | 25.5 |  | 0.69 | 0.70 | 0.21 |
| rs12473679 | C/T | 47.8 |  | 0.58 | 0.87 | 0.40 |  | 45.1 |  | 0.70 | 0.69 | 1.00 |
| rs17529497 | A/G | 24.7 |  | 0.24 | 0.37 | 0.47 |  | 24.7 |  | 1.00 | 0.68 | 0.73 |
| rs7605378 | C/A | 49 |  | 0.49 | 0.75 | 0.57 |  | 49.9 |  | 0.88 | 0.14 | 0.09 |
| rs10203122 | T/C | 31.6 |  | 0.15 | 0.15 | 1.00 |  | 29.3 |  | 0.35 | 0.07 | 0.31 |
